# Tenecteplase versus alteplase for patients with acute ischemic stroke: a meta-analysis of randomized controlled trials

**DOI:** 10.18632/aging.205315

**Published:** 2023-12-26

**Authors:** Xu Zhang, Teng-Fei Wan, Jing Chen, Liang Liu

**Affiliations:** 1 Department of Cardiac Surgery, The General Hospital of Northern Theater Command, Shenyang, Liaoning 110000, China; 2 Department of Nursing, Xinqiao Hospital, Chongqing 400037, China; 3 Department of Critical Care Medicine, The General Hospital of Northern Theater Command, Shenyang, Liaoning 110000, China; 4 Department of Neurology, Central Hospital of Baoji, Baoji, Shaanxi 721000, China; 5 Department of Neurology, The General Hospital of Northern Theater Command, Shenyang, Liaoning 110016, China

**Keywords:** acute ischemic stroke, tenecteplase, alteplase, intravenous thrombolysis, meta-analysis

## Abstract

Tenecteplase (TNK), a newer fibrinolytic agent with greater fibrin specificity and longer half-life than alteplase, may has practical advantages over alteplase in acute ischemic stroke (AIS) thrombolysis. We aimed to perform a systematic review and meta-analysis of randomized controlled trials (RCTs) to compare different doses of TNK (0.1, 0.25, 0.4 mg/kg) and alteplase in acute ischemic stroke patients. We systematically searched PubMed, Embase and https://clinicaltrials.gov/ for RCTs comparing TNK with alteplase in this population eligible for thrombolysis. The Cochrane Risk of Bias Tool was used to assess study quality. Random-effects or fixed-effects meta-analysis models were used for evaluating all outcomes. Total 10 RCTs with 5097 patients were included. Compared with alteplase, TNK at doses of 0.25 mg/kg may associated with the greatest odds to achieve 90-day excellent independence (mRS score ≤1), but there were no significant differences between other doses of TNK (0.1 mg/kg and 0.4 mg/kg) and alteplase. Among secondary outcomes, no significant differences were found in functional outcome (mRS score ≤2) and mortality at 90 days between any dose of TNK and alteplase. Compared with alteplase, TNK was effective at doses of 0.1 mg/kg and 0.25 mg/kg without increased risk of symptomatic intracerebral hemorrhage (sICH), but patients treated with TNK 0.4 mg/kg showed increased odds of sICH. In conclusion, compared with alteplase, intravenous thrombolysis with TNK at dose of 0.25 mg/kg has a better efficacy and similar safety profile and is a reasonable option for patients with AIS.

## INTRODUCTION

Intravenous thrombolysis with alteplase is a standard therapy for patients who present with ischemic stroke symptoms within the first 4.5 h of onset in guidelines [1]. Tenecteplase (TNK), which is modified variant form alteplase by genetic recombinant technology, with greater fibrin specificity and a longer serum half-life is administered as a single bolus rather than as an infusion [2, 3]. Accumulating evidences from clinical trials has suggested benefits of TNK compared with alteplase for AIS [4–6]. A meta-analysis for fourteen studies, including retrospective trials and randomized controlled trials (RCTs), suggested that TNK is as safe as alteplase and may provide more effectiveness than alteplase in AIS patients [7]. Additionally, previous meta-analysis of 5 RCTs indicated that TNK is as safe and effective to alteplase in the intravenous thrombolysis treatment for AIS [8]. However, the safe and effective dose of TNK for patients with AIS still has not been determined clearly yet. Recently, a randomized trial found that compared with alteplase, treated with TNK at a dose of 0.4 mg/kg result in worse functional and safety outcomes for patients with AIS [9]. The controversy led to several RCTs have been conducted recently. Thus, we intend to conduct a pooled analysis of data from newly published RCTs to compare the efficacy and safety of TNK at different doses (0.1, 0.25, 0.4 mg/kg) with alteplase for the intravenous thrombolysis treatment of AIS patients in this study.

## METHODS

This study was performed following the prespecified protocol according to the PRISMA (Preferred Reporting Items for Systematic Reviews and Meta-Analyses) guidelines [10].

### Data sources, inclusion and exclusionE criteria

Two investigators (X.Z., T.-F.W.) performed the literature search independently in PubMed, Embase, and ClinicalTrials.gov inception to April 17, 2023, using search strategy “alteplase” and “tenecteplase” and “stroke”. Only studies published in English language were searched. To avoid missing eligible studies, we retrieved all of the articles and also manually checked the references. The inclusion criteria were as follows: (1) randomized clinical trial; (2) allocation to TNK versus alteplase; (3) compared outcomes of TNK vs. alteplase for patients with AIS; (4) efficacy outcomes were analyzed by modified Rankin scale (mRS); (5) patients (aged ≥18 years) enrolled with acute ischemic stroke (in the anterior circulation or posterior circulation, with a premorbid score of two or less on the mRS also were included); (6) all the participants could be treated with TNK or alteplase within 6 hours after symptom onset. Studies with unavailable data to get effect estimates were excluded. Non-English-language studies, non-RCTs, duplicate reports, commentaries, fundamental experiment studies, abstracts, meeting proceedings were also excluded. To determine whether the data presented in the articles had been duplicated in other publications, we checked the data presented in the articles carefully and we excluded the subgroup analysis studies.

### Data extraction and outcomes

Data were extracted and documented by two authors independently (T.-F.W., L.L.). Details recorded from each study include the name of study, study period, sources of data, sample size, TNK dose(s), and primary outcome. Any disagreements were resolved by consensus review.

The primary efficacy end point was excellent functional outcome (defined as patients who had a score of 0 or 1 on the mRS or a return to baseline for patients with a pre-stroke and the mRS score was 2) at 90 days. The mRS score is a 7-point ordered categorical scale from 0 to 6 for functional neurological outcome, with a lower score indicating independent living and 6 indicating death. Secondary outcomes were the following: good functional outcome at 90 days (defined as a mRS ≤2), symptomatic intracranial hemorrhage (sICH, according to the definition used in each study) and mortality at 90 days.

### Quality assessment

Two authors (T.-F.W., L.L.) conducted the quality assessment of this study independently. We evaluated the risk of bias of all of included RCTs according to the Cochrane Collaboration’s tool and the Jadad Scale [11, 12]. The following seven domains were evaluated based on Cochrane Collaboration’s tool, including sequence generation (selection bias), allocation concealment (selection bias), blinding of participants and personnel (performance bias), blinding of outcome assessors (detection bias), incomplete outcome data (attrition bias), reporting biases (reporting bias), and other potential sources of bias. According to established criteria, the risk of bias in each domain would be scored low, unclear, or high. The study was defined as having a high (>4 high-risk domains), moderate (2–3 high-risk domains) or low (0–2 high-risk domains) risk of bias.

### Statistics

Data synthesis and statistical analysis were conducted by STATA software, version 12.0 (StataCorp, USA). The results of meta-analysis were expressed as odds ratios (ORs) with corresponding 95% confidence intervals (95% CIs). The random-effects (DerSimonian-Laird method) or fixed-effects (Mantel-Haenszel method) meta-analysis model was used to calculate the results, and which meta-analysis model would be used in line with the heterogeneity among the included RCTs. Equivalent Z test was used to determine the statistical significance of pooled ORs and 95% CIs [13]. The heterogeneity among the included RCTs was evaluated using the *P* value of Chi-square-based Q-tests and the I-squared (*I*^2^) statistic [14]. Publication bias of included RCTs was evaluated using the Egger regression method [15], and showed by funnel plots. *P* value less than 0.05 was defined to have a statistical significance.

### Availability of data and materials

The datasets used and/or analyzed during the present study are available from the corresponding author on reasonable request.

## RESULTS

### Study selection and study characteristics

**Figure 1:**
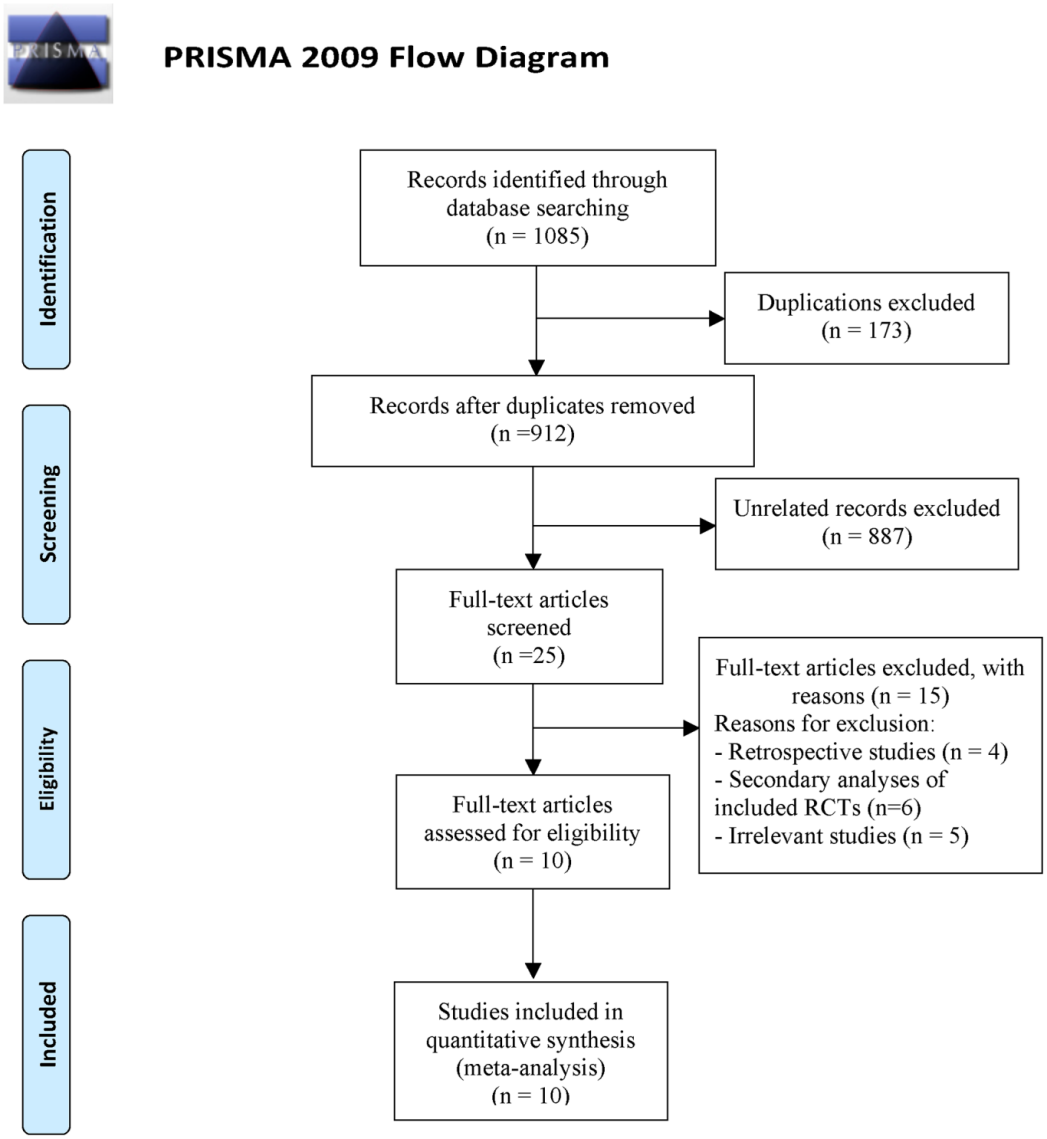
Flowchart of literature search and study selection.

We identified 1085 potentially relevant citations from PubMed, Embase and https://clinicaltrials.gov/ (Figure 1). Of these, 173 duplicated articles were excluded. And we screen abstract and title to exclude 887 unrelated articles. Among the remaining 25 studies, 15 studies were excluded after read the full article and 10 studies with 5097 patients met the selection criteria [4, 5, 9, 16–22]. All eligible RCTs were conducted between 2006 and 2022. In eight of the trials, intravenous thrombolysis was conducted within 4.5 h of symptom onset while in a trial it was within 6 h, and one trial it was within 3 h. Patients were randomly assigned to TNK (at doses of 0.1, 0.25, 0.32 mg/kg) or alteplase at a dose of 0.9 mg/kg. All of the included studies had evaluated the mRS score at 90 days after treatment. The characteristics of included RCTs in this study were presented in Table 1.

**Table 1:** Characteristics of studies included in meta-analysis.

**Study**	**Study period**	**Country**	**Patients, *n***	**TNK dose(s), mg/kg**	**Primary outcome**
TNK Phase IIB	2006–2008	United States	112	0.1/0.25/0.4	Functional Handicap (mRS) at 90 days
Australian TNK	2008–2011	Australia	75	0.1/0.25	Achieving functional independence (mRS score, 0–2) at 90 days
ATTEST	2012–2013	Scotland	96	0.25	Favorable outcome defined as mRS score 0–2 at 90 days after stroke onset
NOR-TEST	2012–2016	Norway	1100	0.4	Excellent (mRS: 0–1 points) functional outcome at 3 months
EXTEND-IA TNK	2015–2017	Australia and New Zealand	202	0.25	Reperfusion of greater than 50% of the involved ischemic territory or an absence of retrievable thrombus at the time of the initial angiographic assessment.
TRACE	2018–2020	China	236	0.1/0.25/0.32	Improvement on NIHSS of ≥4 points or a score ≤1 at day 14
NOR-TEST 2, part A	2019–2021	Norway	204	0.4	A favourable functional outcome at 3 months, defined as an mRS score of 0–1 or a return to baseline if the prestroke mRS score was 2.
TASTE-A	2019–2021	Australia	104	0.25	The volume of the perfusion lesion on CT-perfusion imaging performed on arrival at the receiving hospital
AcT	2019–2022	Canada	1567	0.25	A score of 0 or 1 on the mRS at 90 days, up to 120 days after randomisation.
TRACE-2	2021–2022	China	1401	0.25	Excellent (mRS: 0–1 points) functional outcome at 3 months

Abbreviations: TNK: tenecteplase; mRS: modified Rankin Scale; CT: computed tomography.

### Quality assessment and publication bias

Overall, the quality of included RCTs were assessed with a low risk of bias (Additional file 1: Supplementary Table 1) according to the Jadad scale and Cochrane Risk of Bias Tool. In the domain of the blinding of participants and personnel, all of the included RCTs showed a high risk of bias for these included trials were blinded for outcomes assessor but not researchers and participants. No significant publication bias was found in the included RCTs (Egger’s test: *P* = 0.402, Supplemental Figure 1).

### Primary outcome

All of the included trials with 5097 patients were included to analysis the primary outcome of excellent outcome (mRS score ≤1) at 90 days after treatment. Significantly different results were found in primary outcome to favor the TNK at 0.25 mg/kg (OR, 1.21 (95% CI, 1.04–1.54); *P* = 0.014), but 0.1 mg/kg (OR, 0.91 (95% CI, 0.54–1.54); *P* = 0.734) and 0.32 mg/kg (OR, 0.75 (95% CI, 0.39–1.45); *P* = 0.393) TNK groups compared with alteplase, respectively (Table 2). The *I*^2^ values were estimated as 0 both in 0.1 mg/kg and 0.25 mg/kg TNK groups, which indicate no obvious heterogeneity were found. Obvious heterogeneity (*I*^2^ = 73.7%) was observed in 0.4 mg/kg TNK groups, so the random-effect model was performed.

**Table 2:** Summary of pooled analyses for primary and secondary outcomes.

**Outcomes**	**TNK dose**	**Studies**	**Test of association**				**Heterogeneity**		
			**OR (95% CI)**	***P* value**	**Model**	***Z***	***Χ* ^2^**	***P* value**	***I*^2^ (%)**
**Primary outcome**									
Excellent outcome	0.1 mg	3	0.91 (0.54–1.54)	0.734	FE	0.34	0.26	0.880	0.0%
	0.25 mg	8	1.21 (1.04–1.54)	0.014	FE	2.47	5.73	0.572	0.0%
	0.4 mg	3	0.75 (0.39–1.45)	0.393	RE	0.85	7.6	0.022	73.7%
**Secondary outcomes**									
Functional independence	0.1 mg	2	1.07 (0.56–2.04)	0.839	FE	0.20	1.53	0.217	34.4%
	0.25 mg	7	1.13 (0.98–1.30)	0.091	FE	1.77	9.64	0.141	37.8%
	0.4 mg	2	0.65 (0.31–1.36)	0.250	RE	1.15	5.23	0.022	80.9%
sICH, *n* (%)	0.1 mg	3	0.81 (0.23–2.87)	0.739	FE	0.33	2.27	0.322	11.7%
	0.25 mg	8	0.98 (0.79–1.20)	0.817	FE	0.23	8.48	0.293	17.4%
	0.4 mg	3	2.23 (1.04–4.80)	0.040	FE	2.05	2.49	0.288	19.6%
Mortality at 90 days, *n* (%)	0.1 mg	3	0.63 (0.23–1.70)	0.359	FE	0.92	2.73	0.255	26.8%
	0.25 mg	8	1.04 (0.68–1.52)	0.946	FE	0.07	2.12	0.908	0.0%
	0.4 mg	3	1.37 (0.56–3.39)	0.494	RE	0.68	5.15	0.076	61.2%

Abbreviations: TNK: tenecteplase; OR: odds ratio; CI: confidence interval; RE: random effects; FE: fixed effects; sICH: symptomatic intracranial hemorrhage.

In subgroup analysis according to geographic regions, we found that compared with alteplase, no significantly different results were found in the primary outcome (mRS score ≤1) to favor the 0.25 mg/kg TNK in different regions (including China, America, Europe and Australia. Additional file 1: Supplementary Table 2).

### Secondary outcomes

The proportion of good outcome (mRS score 0–2) at 90 days after treatment were not significantly different between participants receiving TNK and alteplase (0.1 mg/kg TNK: OR, 1.07 (95% CI, 0.56–2.04); *P* = 0.839; 0.25 mg/kg TNK: OR, 1.13 (95% CI, 0.98–1.30); *P* = 0.091; 0.4 mg/kg TNK: OR, 0.65 (95% CI, 0.31–1.36); *P* = 0.250) (Table 2). No treatment group differences were found in the incidence of sICH between 0.1 mg/kg or 0.25 mg/kg TNK groups and the alteplase group (0.1 mg/kg TNK: OR, 0.81 (95% CI, 0.23–2.87); *P* = 0.739; 0.25 mg/kg TNK: OR, 0.98 (95% CI, 0.79–1.20); *P* = 0.817) (Table 2). However, it may be important to note that patients receiving TNK at a dose of 0.4 mg/kg with significantly higher incidence of sICH compared with alteplase group (OR, 2.23 (95% CI, 1.04–4.80); *P* = 0.040) (Table 2). Moreover, no significant differences were found between the groups in the rates of 90-day mortality (0.1 mg/kg TNK: OR, 0.63 (95% CI, 0.23–1.70); *P* = 0.359; 0.25 mg/kg TNK: OR, 1.04 (95% CI, 0.68–1.52); *P* = 0.946; 0.4 mg/kg TNK: OR, 1.37 (95% CI, 0.56–3.39); *P* = 0.494) (Table 2).

## DISCUSSION

In this meta-analysis of 10 RCTs, we comprehensively compared the effectiveness and safety between TNK and alteplase in patients with AIS eligible for thrombolysis. There was a significant difference were found in excellent outcome (mRS ≤ 1) as compared TNK at dose of 0.25 mg/kg with alteplase but TNK at dose of 0.1 mg/kg or 0.4 mg/kg. However, no significant differences were found in the rates of functional independence (mRS 0–2), or mortality between patients receiving TNK and alteplase. Moreover, compared with alteplase, TNK at dose of 0.1 mg/kg and 0.25 mg/kg did not appear to increased risk of sICH. Importantly, we noticed that the dose of 0.4 mg/kg TNK increase the incidence of sICH for patients with AIS compared with alteplase.

At present, although intravenous thrombolysis with alteplase remains the only approved choice for patients with AIS eligible for thrombolysis, current guidelines recommended that TNK might be considered as an alternative to alteplase [1, 23]. Our findings are in line with evidences from previous meta-analyses of RCTs, suggesting that no differences are found between any dose of TNK and alteplase for functional independence (0–2) and mortality at 90 days in patients with AIS [8, 24]. However, previous meta-analyses included fewer trials and comparisons of different dose tiers were very limited. In this study, total 10 RCTs were included and is the first to demonstrate that the dose of 0.4 mg/kg TNK appears unsafe for patients with AIS compared with alteplase when the accumulated evidences were collated. Importantly, our results showed that compared with alteplase, TNK at doses of 0.25 mg/kg may associated with the higher odds to achieve 90-day excellent independence (mRS score ≤1). Meanwhile, compared with alteplase, the pooled results from nonrandomized trials found that intravenous thrombolysis with TNK was associated with higher odds of good functional outcome (mRS 0–2) and early neurologic improvement for patients with AIS [9]. In addition, previous meta-analysis which included RCTs and nonrandomized trials showed that TNK might improve early neurologic function compared with alteplase for patients with AIS [7, 25]. Consistently, no statistical differences were found between the TNK and alteplase groups in the proportion of 3-month good functional outcome, sICH and mortality in these previous meta-analyses. Given the fact that absence of randomization and selection bias would be confounded the results of these studies, pooled data from RCTs in our study provided further evidences to resolve this controversy.

Although 0.9 mg/kg was the standard dose of intravenous alteplase in all studies, the dose of intravenous TNK was varied (0.1 mg/kg, 0.25 mg/kg, 0.32 mg/kg and 0.4 mg/kg) and has not been clearly determined. Total 8 RCTs included in this meta-analysis have compared TNK at dose of 0.25 mg/kg with alteplase in patients with AIS, and pooled results provide robust evidences for the comparative efficacy and safety of TNK at a dose of 0.25 mg/kg. Previous network meta-analysis, which found that TNK at a dose of 0.25 mg/kg showed better efficacy and imaging-based outcomes without increased risk of safety outcomes [26]. Moreover, the results of TRACE trial also showed that the 0.25 mg/kg dose tier showed better on excellent functional outcomes than 0.1 mg/kg and 0.32 mg/kg of TNK groups [19]. Among AIS patients with large vessel occlusion whom endovascular treatment is planned, EXTEND-IA TNK Part 2 trial demonstrated that TNK at a dose of 0.4 mg/kg did not provide any additional benefits compared with 0.25 mg/kg [27]. Importantly, NOR-TEST 2 part A trial was designed to demonstrate the non-inferiority of TNK at a dose of 0.4 mg/kg to alteplase for patients with AIS, but was stopped early for safety reasons [9]. This prematurely terminated study found that TNK at a dose of 0.4 mg/kg resulted in worse safety and less frequency of good functional outcomes compared with alteplase. Given the above evidences, TNK at a dose of 0.25 mg/kg might reduce risk of bleeding compared with the dose of 0.4 mg/kg, and showed better outcomes compared with a dose of 0.1 mg/kg, which could be the dose of choice for patients with AIS eligible for intravenous thrombolysis. Especially, CERTAIN trial has found that patients with AIS intravenous thrombolysis with TNK at a dose of 0.25 mg/kg showed lower risk of sICH than alteplase [28]. Currently, an ongoing trial, ATTEST 2 (ClinicalTrials.gov number, NCT02814409), were set to compare 0.25 mg/kg of TNK with alteplase 0.9 mg/kg with excellent functional outcome, which could provide further insight into the efficacy and safety of TNK at a dose of 0.25 mg/kg.

We must acknowledge that this study has limitations. First, the dose of intravenous TNK varied both within and between studies (0.1 mg/kg, 0.25 mg/kg, 0.32 mg/kg and 0.4 mg/kg). Fewer trials have evaluated the dose of 0.1 and 0.4 mg/kg of TNK, compared with the dose of 0.25 mg/kg. Thus, power to detect differences was constrained for 0.1 and 0.4 mg/kg dose. Second, we could not conduct some subgroup analysis for the lack of some of essential data in part of included trials (e.g., baseline NIHSS scores, occlusion site, cause of stroke, stroke onset to needle time, endovascular thrombectomy alone or combined IVT and endovascular thrombectomy and etc.). Third, the description of all adverse reactions mentioned in the included articles were various. Thus, we just analyzed the risk of sICH after intravenous thrombolysis with TNK or alteplase. Lastly, the time window for intravenous thrombolysis varied between studies, including patients with AIS eligible for intravenous thrombolysis within the first 3.0, 4.5 or 6.0 hours from stroke onset. Only one study which has reported the safety and efficacy of intravenous thrombolysis with TNK outside 4.5 hours from stroke onset [5]. Currently, three ongoing trials (TIMELESS trial: NCT03785678 [29], TEMPO-2 trial: NCT02398656 and CHABLIS-T: NCT04086147) are intended to investigate the efficacy and safety of TNK in patients with AIS in extended time window.

## CONCLUSIONS

The pooled data from RCTs in this study provides supporting evidences that compare with alteplase, intravenous thrombolysis with TNK at dose of 0.25 mg/kg shows a better safety and similar efficacy profile and is a reasonable option for patients with AIS. Given the TNK at a dose of 0.4 mg/kg appears unsafe versus alteplase, a lower dose of TNK should be used for patients with AIS. However, these findings are mainly based on smaller sample size and due to several limitations of this study, additional multi-center RCTs to definitively address these issues are warranted.

## Supplementary Materials

Supplementary Figure 1

Supplementary Tables
