# Supplementary figures and images for: Tenecteplase versus alteplase for patients with acute ischemic stroke: a meta-analysis of randomized controlled trials
Source: Aging (Albany NY). 2023 Dec 26;15(24):14889–99. doi: 10.18632/aging.205315 (PMC10781500; doi:10.18632/aging.205315)

SUPPLEMENTARY FIGURE

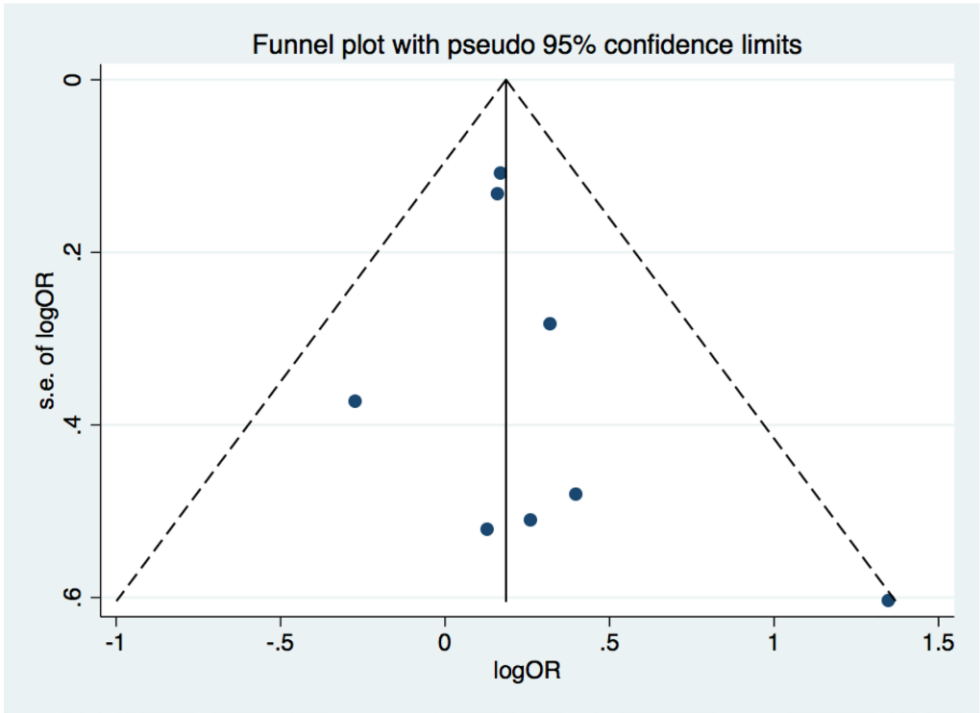

Supplemental Figure 1. Funnel plots of all the included studies.

Supplement: Supplementary Figure 1 [file aging-15-205315-s001.pdf]
