# Supplementary material for: Tenecteplase versus alteplase for patients with acute ischemic stroke: a meta-analysis of randomized controlled trials
Source: Aging (Albany NY). 2023 Dec 26;15(24):14889–99. doi: 10.18632/aging.205315 (PMC10781500; doi:10.18632/aging.205315)
Supplement: Supplementary Tables [file aging-15-205315-s002.pdf]

SUPPLEMENTARY TABLES

Supplementary Table 1. Assessment of the methodological quality of included randomized trials using the cochrane collaboration's tool and the jadad scale.

Trials	Random sequence generation (selection bias)	Allocation concealment (selection bias)	Blinding of participants, personnel (performance bias)	Blinding of outcome assessment (detection bias)	Incomplete outcome data (attrition bias)	Selective reporting (reporting bias)	Other sources of bias	Jadad Score
TNK Phase IIB	Low bias	Low bias	Low bias	Low bias	Low bias	Low bias	Unclear	5
Australian TNK	Low bias	Unclear	High bias	Unclear	Low bias	Low bias	Low bias	3
ATTEST	Low bias	Low bias	High bias	Unclear	Low bias	Low bias	Low bias	3
NOR-TEST	Low bias	Unclear	High bias	Unclear	Low bias	Low bias	Low bias	3
EXTEND-IA TNK	Low bias	Low bias	High bias	Unclear	Low bias	Low bias	Unclear	3
TRACE	Low bias	Low bias	High bias	Low bias	Low bias	Low bias	Unclear	3
NOR-TEST 2, part A	Low bias	Low bias	High bias	Low bias	Low bias	Low bias	Unclear	3
TASTE-A	Low bias	Low bias	High bias	Low bias	Low bias	Low bias	Unclear	3
AcT	Low bias	Low bias	High bias	Low bias	Low bias	Low bias	Unclear	3
TRACE-2	Low bias	Low bias	High bias	Low bias	Low bias	Low bias	Unclear	3

Supplementary Table 2. Summary of subgroups meta-analysis results according to geographic regions.

Regions	Studies	Test of association		
		OR (95% CI)	*P* value	*Z*
China	2	1.06 (0.97–1.15)	0.197	1.29
America	2	1.29 (0.99–1.66)	0.193	1.30
Europe	1	1.36 (0.66–2.79)	0.408	0.83
Australia	3	1.29 (0.99–1.66)	0.056	1.91
